# Biotechnological Potential of *Bacillus salmalaya* 139SI: A Novel Strain for Remediating Water Polluted with Crude Oil Waste

**DOI:** 10.1371/journal.pone.0120931

**Published:** 2015-04-13

**Authors:** Salmah Ismail, Arezoo Dadrasnia

**Affiliations:** Department of Biohealth Science, Institute of Biological Sciences, Faculty of Science, University of Malaya, Kuala Lumpur, Malaysia; Nanyang Technological University, SINGAPORE

## Abstract

Environmental contamination by petroleum hydrocarbons, mainly crude oil waste from refineries, is becoming prevalent worldwide. This study investigates the bioremediation of water contaminated with crude oil waste. *Bacillus salamalaya* 139SI, a bacterium isolated from a private farm soil in the Kuala Selangor in Malaysia, was found to be a potential degrader of crude oil waste. When a microbial population of 10^8^ CFU ml^-1^ was used, the 139SI strain degraded 79% and 88% of the total petroleum hydrocarbons after 42 days of incubation in mineral salt media containing 2% and 1% of crude oil waste, respectively, under optimum conditions. In the uninoculated medium containing 1% crude oil waste, 6% was degraded. Relative to the control, the degradation was significantly greater when a bacteria count of 99 × 10^8^ CFU ml^-1^ was added to the treatments polluted with 1% oil. Thus, this isolated strain is useful for enhancing the biotreatment of oil in wastewater.

## Introduction

During the transport and production of crude oil and its derivatives, leakage and unsuitable operations may result in environmental contamination by petroleum hydrocarbons. Contamination by petroleum hydrocarbons is an important issue in environmental research because petroleum hydrocarbons can significantly affect the environment and pose a serious and substantial hazard to human health [1]. Aquatic oil pollution is a serious global threat. The inputs from natural marine oil seeps alone could cover the world's oceans in a layer of oil that is 20 molecules thick [2]. Globally, the total amount of petroleum hydrocarbon released into the sea and ocean from all sources is approximately 1.3 Mt per year [3]. Currently, various cleanup techniques are available, including soil vapor extraction, solidification and soil washing. Compared to chemical and physical methods (oxidants, adsorbents, chemical surfactants, etc.), which are expensive and require further treatment or disposal of the incinerated soil, bioremediation is an eco-friendly, economical and self-driven method. Highly hazardous oily materials can be easily mineralized to harmless products using suitable microorganisms [4]. Indigenous microorganisms in the environment can degrade a wide range of organic compounds; however, their activities and populations are affected by high concentrations of oil [5,6]. Recently, the potential of a microbe-produced surfactant as a natural compound to reduce the cell surface and increase the rate of organic compound degradation has been explored. Biosurfactants are widely applied for environmental protection to enhance oil recovery and tanker oil spill cleanups and to remediate soils contaminated with heavy metals and hydrocarbons. Many reports have described the efficiency of biosurfactants and additional microorganisms for biodegrading and enhancing hydrocarbon bioremediation [3,7–9]. Accordingly, this study aims to report and evaluate the biotechnological potential of a novel species strain from the genus *Bacillus* (identified by the complete 16S rRNA gene (*Bacillus salmalaya* 139SI) sequence) in the degradation of crude oil waste in water.

## Materials and Methods

### Isolation and screening of the strain

Strain Salmah Ismail (SI) 139SI was originally isolated from soil obtained from the private farm in Selangor, Malaysia (2.99917°N 101.70778°E) [10]. The obtained soil sample was suspended in 3 ml of sterile distilled water, and the suspension was later streaked on brain-heart infusion (BHI) agar plates that were supplemented with 5% sheep blood. The plates were incubated for 16–24 h at 37°C. Hemolytic colonies were isolated from the plates and sub-cultured on BHI blood agar. The sub-cultured plates were incubated for 16–24 h at 37°C under aerobic conditions. The 139SI strain was one isolate that exhibited strong hemolytic activity. Thus, this strain was routinely cultured on BHI blood agar and was maintained in a glycerol suspension (25%, w/v) at -80°C and on BHI-slant agar at room temperature.

### 16S rRNA sequencing identification

The 16S rRNA sequencing and phylogenetic analysis of strain 139SI (GenBank accession No: JF825470.1; ATCC BAA-2268) were conducted using three strains of *Bacillus* isolates that were selected as the type strains of the proposed novel species. Molecular identification was performed by extracting the genomic DNA (with 27F and 1492R primers) using a Bactozol Kit. The extracted DNA was purified using a QIAGEN column. The 16S rRNA gene sequence for strain 139SI was compared with the available 16S rRNA gene sequences from GenBank using the BLAST program (http://www.ncbi.nlm.nih.gov/BLAST/). The phylogenetic position was determined for strain 139SI based on 16S rRNA gene sequences.

### Preparation of strain 139SI cell-free supernatant

In order to identify and analyze the properties of the isolate, preparation of a pure culture is essential and required. This is achieved by using an enrichment media that can provide favorable growth conditions for the organisms of interest. Therefore, an overnight culture of strain 139SI was transferred into centrifuge tube under sterile conductions and the culture was centrifuged at 14300 xg at 4°C for 20 min to separate the cell from the supernatant. The obtained supernatant was subjected to sterile filtration to which is considered the optimal method to remove all particles and dead microorganisms without any influence of their ingredients, therefore used syringe filter with a pore size of 0.22 μm for that purpose to yield a purified cell-free supernatant (CFS) to be assayed later for this study.

### Haemolytic activity and antibiotic resistance of strain 139SI

Strain 139SI was screened on blood/agar plates containing 5% (v/v) blood and incubated at 37°C for 48 h. Haemolytic activity was detected as the presence of a definite clear zone around a colony. Minimum inhibition concentration value to Ciprofloxacin, Amikacin, Doripenem, Ampicilin, Linezolid and Aztreonam was determinate on Trypticase soy agar plates using MIC trat strip antibiotic sensitivity test. Zone of inhibition was noted after 24 h of incubation. The result was compared with the European committee on antimicrobial susceptibility testing (EUCAST) database.

### Determination of biofilm inhibitory concentration (BIC)

In order to determine the lowest concentration of 139SI filtrate that can cause visible inhibition in the biofilm formation and significant reduction in the readings when compared with the control wells, the BIC test was performed as described previously [11]. The clinical bacterial isolate that was selected for the BIC test was a resistant *Pseudomonas aeruginosa* isolate recovered from a patient with chronic tonsillitis. A piece of glass cover slip placed inside each of the wells to allow the growth and visualization the effect of filtrate on the tested bacterial isolate. About 300 ml of sterile BHI broth into each well of the microtiter plate (MTP) and inoculated it with 160 ml overnight culture of *P*. *aeruginosa* followed by addition of 160 ml of various concentrations of soil bacterial filtrate (1000–5000 μg/ml). Plates were incubated for 24 h at 37°C and the biofilm inhibition was determined spectrophotometrically via MTP reader.

### Cell hydrophobicity and emulsification index (E_24_)

Strain 139SI was enriched in 20 ml MSM and supplemented with 2% (w/w) crude oil for one week at 33°C in an orbital shaker at 5 xg. Cells were harvested by centrifugation and resuspended in sterile MSM. The OD_600_ of the cell suspension was adjusted to approximately 0.5. Then, 200 μl of oil was added to the cell suspension, and the suspension was vortexed for 3 min. Changes in the OD_600_ were recorded for the cell suspension after allowing the oil and aqueous phase to separate. The cell hydrophobicity was expressed as cell adherence (%) to the oil, which was calculated as shown below.

BA(%)=(1−Dα/Dβ)×100 (1)

where BA, Dβ and Dα represent the Bacterial adherence, absorbance of the cell suspension before and after shaking with oil, respectively. The emulsification index at 25°C was determined by adding 4 ml of crude oil to 4 ml of aqueous phase containing the biosurfactant. Next, the suspension was vortexed for 5 min. After 24 h, the total heights of the oil phase, the aqueous phase and the emulsified layer were recorded. The emulsification index was calculated as follows:

E24(%)=He/Ht×100 (2)

where H_e_ and H_t_ represent the heights of the emulsified layer and the total height, respectively.

### Optimization of environmental factors on *Bacillus salmalaya* 139SI

The growth of strain 139SI was evaluated under different environmental conditions (pH and temperature) by inoculating 20 ml of autoclaved mineral salt medium (MSM) containing 2% crude oil with the bacterial strain. The flasks were incubated at 5 xg and 35°C for one week. In addition, growth was monitored at different pHs (4, 5, 6, 7, 8 and 9) and temperatures (25°C, 30°C, 35°C and 40°C).

### Biodegradation of crude oil waste

Strain 139SI was enriched in 100 ml of BHI broth in flasks (250 ml) that were placed on an orbital shaker at 5 xg and 37°C. The incubation continued until the OD_600_ equaled 1. The production of biosurfactant in BHI media prepared using 10% (w/v) of seed culture. Bacterial cells were harvested by centrifugation at 4°C (5600 xg). Next, the harvested cells were resuspended in sterilized MSM. The biodegradation of oil by this strain was conducted in 250 ml of sterilized MSM (pH = 7) with 1% and 2% (w/v) of crude oil waste. Finally, inoculum was added separately at 2% (w/v) to each of the above media. The experiment was performed using three replicates of each treatment. The inoculated medium was the control treatment. The flasks were incubated in an incubator shaker maintained at 35°C and 5 xg for 42 days. Every seven days, three flasks for each organism and the control were removed from the incubator shaker. The residual oil was extracted twice using 100 ml of solvent and dried with anhydrous sodium sulfate. The solvent was removed using a rotary evaporator, and the weight of the residual oil was measured and recorded. The percentage of oil degradation was calculated using the following formula:

Biodegradation(%)=TC−TT/TC×100 (3)

where TPH is the total petroleum hydrocarbon, TC is the TPH in the control, and TT is the TPH in the treatment. The residual hydrocarbons in the water were determined using GC coupled to a mass spectrometry (MS). The helium carrier gas flow was 1.27 ml min^-1^. The column oven was initially held at 50°C for 2 min, increased to 300°C at a rate of 6°C min^-1^ and then increased to 300°C and held for 16 min.

### Bacterial growth analysis

The colony forming units (CFU) mL^-1^ of the bacteria were determined by counting the colonies (dilution method) on the BHI plates that were incubated at 33°C for 16–24 h.

### Biochemical oxygen demand (BOD)

To assess the indirect capability to degrade pollutants, measurements of the dissolved oxygen (DO) concentration was meastured by a dissolved oxygen meter in accordance with the manufacturer’s instructions.

### Statistical analyses

To evaluate the statistical results, a general linear model (SPSS 18) was used for the ANOVA between the means of the treatments. In addition, Duncan's multiple range test was performed to test for significance (P < 0.05).

## Results and Discussion

### Morphological identification and phylogenetic analyses of 139SI

**Fig 1:**
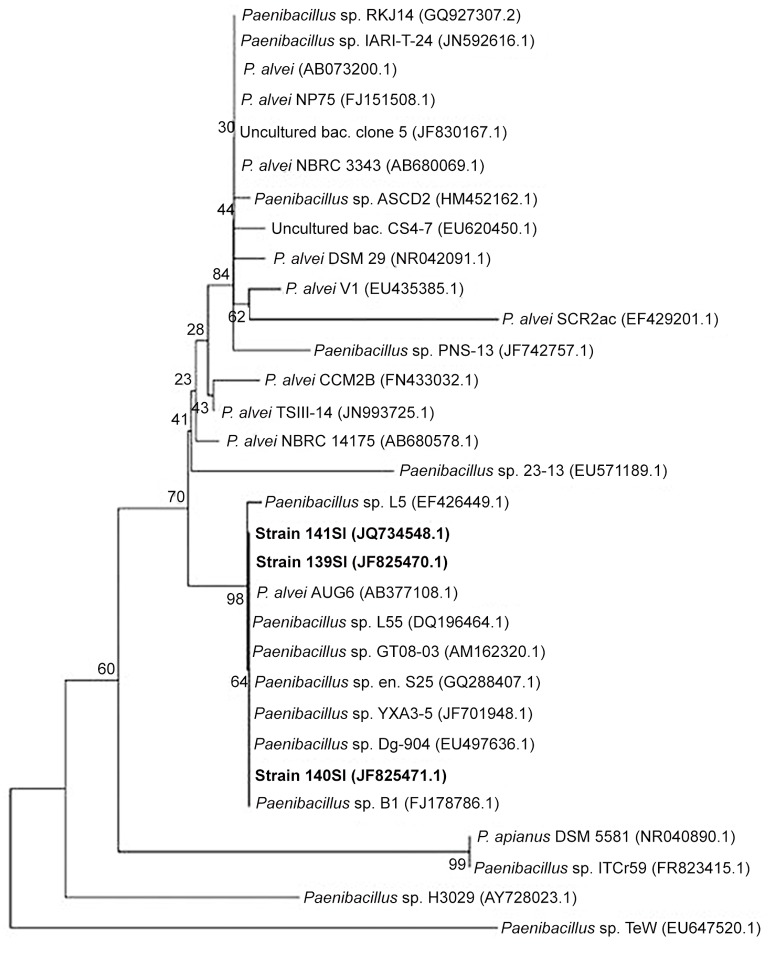
Phylogenetic position based on the 16S rRNA gene sequences. The neighbor-joining method was performed, followed by 1000 replications of bootstrapping.

The isolated colonies were large and gray with a rough and irregular edge. In addition, the colonies were 2–3 mm in diameter and exhibited strong hemolytic activity after 16 h of incubation at 37°C on 5% sheep blood agar. Strains of SI reacted positively to glucose, sucrose and oxidase tests and negatively to urease, indole, lactose and mannitol tests. The optimal growth temperature was 37°C, and no growth was observed at 50°C. Overall, the 16S rRNA gene sequence of strain 139SI was 1496 bp in length. The hypothesis that strain 139SI represents a novel species of the *Bacillus* genus is strengthened by the differences that were observed in the 16S rRNA sequence relative to the recognized species of this genus. The 16S rRNA gene sequence was very similar (99%) to *Paenibacillus alvei* and less similar (95%) to *Paenibacillus terrigena*. In addition, *Paenibacillus* was found in the phylogenetic tree that was obtained using the neighbor-joining method (Fig. 1).

### Haemolytic and antibiotic activity

The isolated colonies were grey, large, rough and irregular edged with a size of 2–3 mm in diameter exhibiting a strong hemolytic activity after 16 h incubation at 37°C on 5% sheep blood agar. In addition, milky smears were observed for the drop collapsing test for biosurfactant production by the crude filtrate from strain 139SI confirming the co-existence of haemolytic activity and biosurfactant production of this bacteria. Biosurfactant-producing capacity in liquid medium was found to be associated with haemolytic activity [12]. Haemolytic activity therefore appears to be a good screening criterion in the search for surfactant-producing strains. Such screening can be used to limit the number of samples that are subsequently subjected to biosurfactant-activity tests in liquid media (S1 Fig). The result of antibiotic resistance illustrated in Table 1. Bacterium was found to be most resistant to Ciprofloxacin, Amikacin, Doripenem, Ampicilin and Linezolid, while it was susceptible to Aztreonam compared with EUCAST database.

**Table 1:** MIC of antibiotics.

Antibiotic name	EUCAST non-species related break-points (μg/ml)		MIC results(μg/ml)
	S ≤	R >	139SI
Ciprofloxacin(CIP)	0.0005	0.001	0.190 (R)
Amikacin (AK)	0.008	0.016	0.50 (R)
Doripenem (DOR)	0.001	0.004	0.06 (R)
Ampicilin (AMP)	0.002	0.008	128.00 (R)
Linezolid (LNZ)	0.002	0.004	2.00 (R)
Aztreonam (ATM)	0.004	0.008	0.00 (S)

EUCAST–European Committee on Antimicrobial Susceptibility Testing

S–Susceptible

R–Resistant

### Biofilm inhibitory concentration

The most effective concentration was 4500μg/ml that resulted in a decreased adherence index when quantified spectrophotometrically. There was a significant decrease in the adherence index among all the isolates tested with variations in the degree of adherence to the surface. The results shows 80% inhibition in the biofilm when visualized under light microscope showing scattered bacterial cells with no extracellular matrix (S2 Fig). However, strain 139SI capable of forming biofilms becomes a potential candidate to be utilized in the process of bioremediation.

### Cell hydrophobicity and emulsification index

The observed cell hydrophobicity of 139SI was 78.5 ± 2. The rate of hydrophobicity was dependent on the composition of the cell surface and the uptake mechanisms of the hydrocarbons. However, the addition of a biosurfactant or chemicals could reduce the degradation rate of organic compound [13]. Norman et al. [14] demonstrated that strains U_1_ and U_3_ were able to reduce their cell hydrophobicity to degrade Bonny light crude oil. The emulsification index of 139SI was 69% ± 1.8. The high emulsification (E_24_) facilitated the bioavailability of the organic compounds for bacteria and resulted in faster hydrocarbon degradation.

### Effects of environmental conditions on strain growth

**Fig 2:**
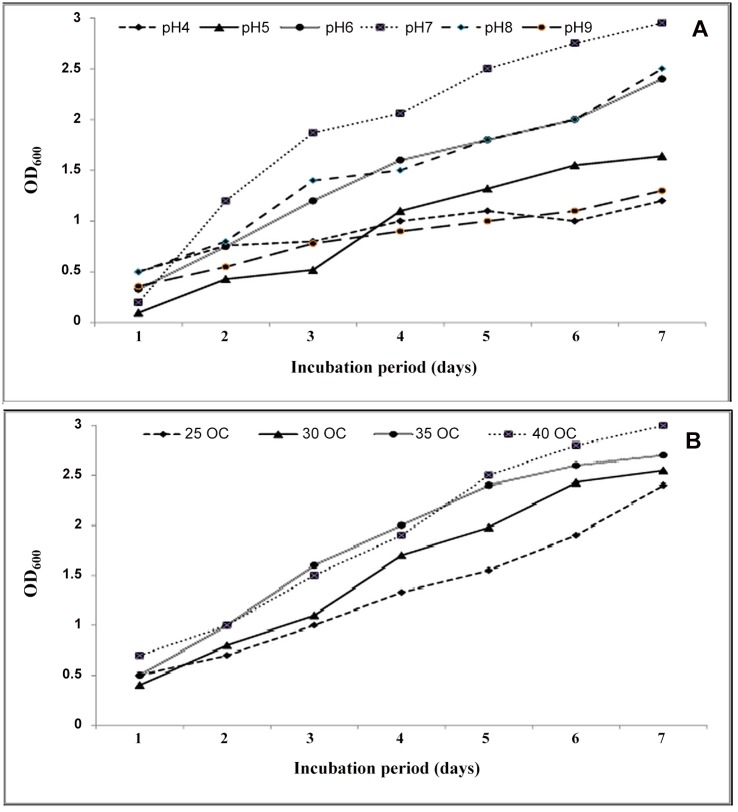
Effect of different pHs (A) and temperatures (B) on the optical density of strian 139SI.

When strain 139SI was grown in MSM with 1% and 2% oil at 5 xg and 35°C, the optical density reached 1.8 after 7 days of incubation. Additionally, the crude oil waste was rapidly used as a source of carbon. In different environmental conditions, the OD trend increased during the 7 days. For a range of pHs, the OD_600_ for 139SI was highest, 2.9, at a pH of 7, followed by pH 8, pH 6 and pH 5 (Fig. 2A). The lowest rates of OD after 7 days were observed at pH 4 and pH 9, with values of 1.2 and 1.3, respectively. Regarding temperature, the highest and lowest OD_600_ values were recorded at 40°C and 25°C, respectively (Fig. 2B). The mineralization and uptake of organic compounds by microorganisms depend on their concentrations and compositions [15]. In addition, biological, chemical and physical conditions affect the survivability and abilities of a strain in a contaminated environment. Kumari et al. [4] observed and recorded the abilities of *Pseudomonas* sp. BP10 and *Rhodococcus* sp. NJ2 that were isolated from crude oil-contaminated sites containing 10% oil. These authors indicated that the isolated BP10 used oil faster, with a use rate of 9 xg at 35°C after 6 days of incubation (OD_600_ = 1.8).

### Biodegradation of crude oil waste

**Fig 3:**
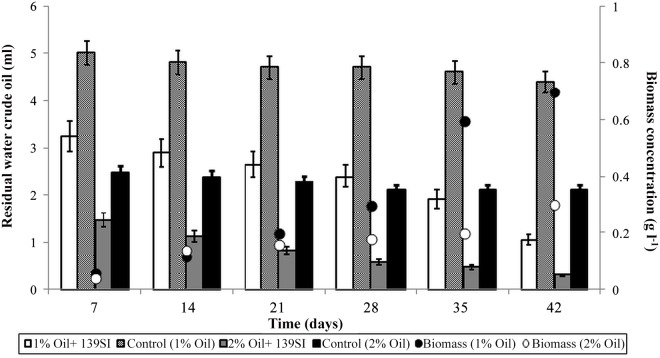
Total residual crude oil waste (bar) and produced biomass concentrations (circle).

The total produced biomass concentrations and degraded oil during the incubation time are summarized in Fig. 3. The maximum biomass growth (700 mg /L) as well as maximum biodegradation percentage (88%) was achieved in treatment polluted with 1% crude oil at the end of study period. Total petroleum hydrocarbons (TPHs) in the crude oil waste that was amended with 139SI were degraded by approximately 79% during the incubation period in the treatments with 2% crude oil. A gravitational analysis of the crude oil waste shows that 3.95 and 2.2 ml of the TPH were reduced after 42 days of incubation in the treatments with 1 and 2% oil, respectively (Fig. 3).

**Fig 4:**
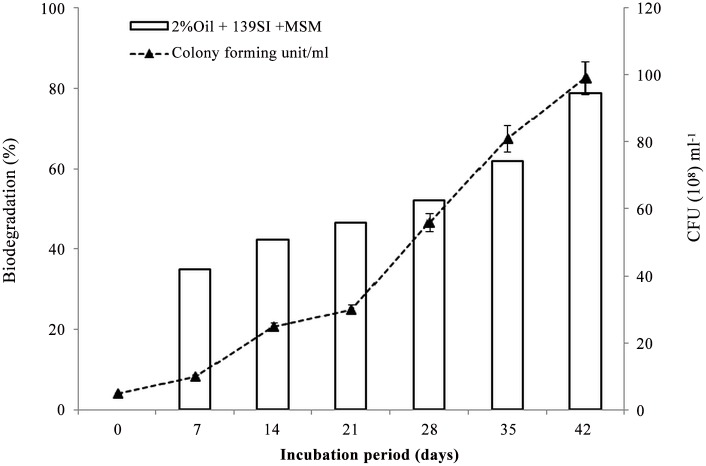
Growth and rate of degradation ability of strain 139SI during the incubation period. MSM: mineral salt medium, CFU: colony-forming unit.

In the control, the TPH was only reduced to 0.4 and 0.6 ml in the treatments with 1 and 2% crude oil waste, respectively. However, the degradation rates in the treatments that were polluted with 1% oil and amended with 139SI were higher than in the treatments polluted with 2% oil (S3 Fig). Finally, the optical density of the culture reached 3.1. The efficient degradation of oil in oil-polluted areas that were affected by microorganisms was previously reported [16–18]. The statistical analysis based on Duncan’s test showed no significant biodegradation of hydrocarbons in the control samples, whereas significantly different TPH degradation occurred between the treatments and the controls. The F-values of the ANOVA between the days and treatments were 44.5 and 32, respectively. In the biodegradation studies, higher F-values were observed between treatments, which indicated that the selected bacteria were effective for oil remediation. In addition, although the number of CFU initially increased slowly, the rate of growth sharply increased after 28 days of incubation. The cell counts of the bacterial strain were 99 ± 2 × 10^8^ CFU/ml at the end of the incubation and 5 ± 0.3 × 10^8^ CFU/ml at the beginning of the incubation (Fig. 4). The increase in microbial population during the remediation process except for the control microcosm was an indication of their ability to utilize petroleum hydrocarbon as a source of energy.

**Fig 5:**
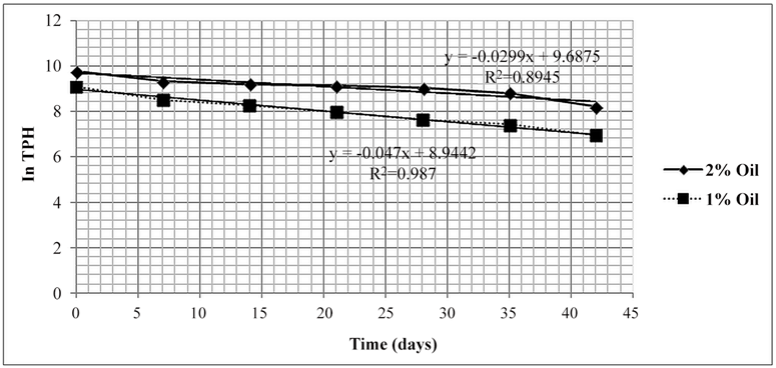
First order graph showing in TPH as crude oil waste against time for the remediation process.

It was obvious that the TPH concentration decreased with time as a result of microbe growth in the broth (Table 2). The regression coefficients (R^2^) obtained from the linear plot were 0.98 and 0.89 in treatments polluted with 1% and 2% crude oil waste, respectively. This suggests very strong relationship between time and the rates of biodegradation of TPH as seen in Fig. 5. However, Fig. 4 is in conjunction with the graph in Fig. 5 graphically satisfied the conditions for first order reaction, with a rate constant of 0.05 and 0.03 day^-1^ and half-life of 13.86 and 19.25 days in treatment polluted with 1% and 2% oil, respectively. Bioremediation efficiency of 88% was achieved as seen in Table 2. This suggests that the bioaugmentation process was effective in clean-up of groundwater contaminated with crude oil waste.

**Table 2:** Data for kinetic studies of remediation.

Time (days)	TPH left	Ln TPH	% Removal
1% oil			
0	8620.6	9	0
7	5086.2	8.5	41
14	3862.06	8.2	55.2
21	2844.8	7.9	67
28	1982.7	7.6	77
35	1637.9	7.4	81
42	1034.4	6.9	88
2% oil			
0	17241.3	9.7	0
7	11241.3	9.3	34.9
14	9965.51	9.2	42.3
21	9206.9	9.1	46.6
28	8275.8	9	52
35	6586.2	8.8	61.8
42	3655.1	8.2	78.8

TPH: total petroleum hydrocarbon

### Biochemical oxygen demand

**Fig 6:**
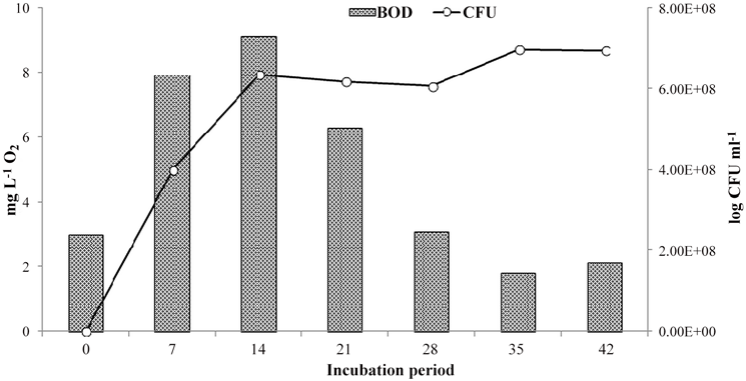
The relation between total bacterial population and BOD.

The consumption of oxygen as indicated by BOD data shows a peak value on the 14^th^ day (Fig. 6). After this day until the end of incubation time BOD value shows decreasing trend. It is interesting to note that the bacteria community increased in abundance after that the biochemical demand for oxygen required by organic matter decomposition decreased. The reason for this trend was that the consumption of O_2_ by the bacteria increased, following the introduction of oil into the samples [19].

### Analysis of residual oil

**Fig 7:**
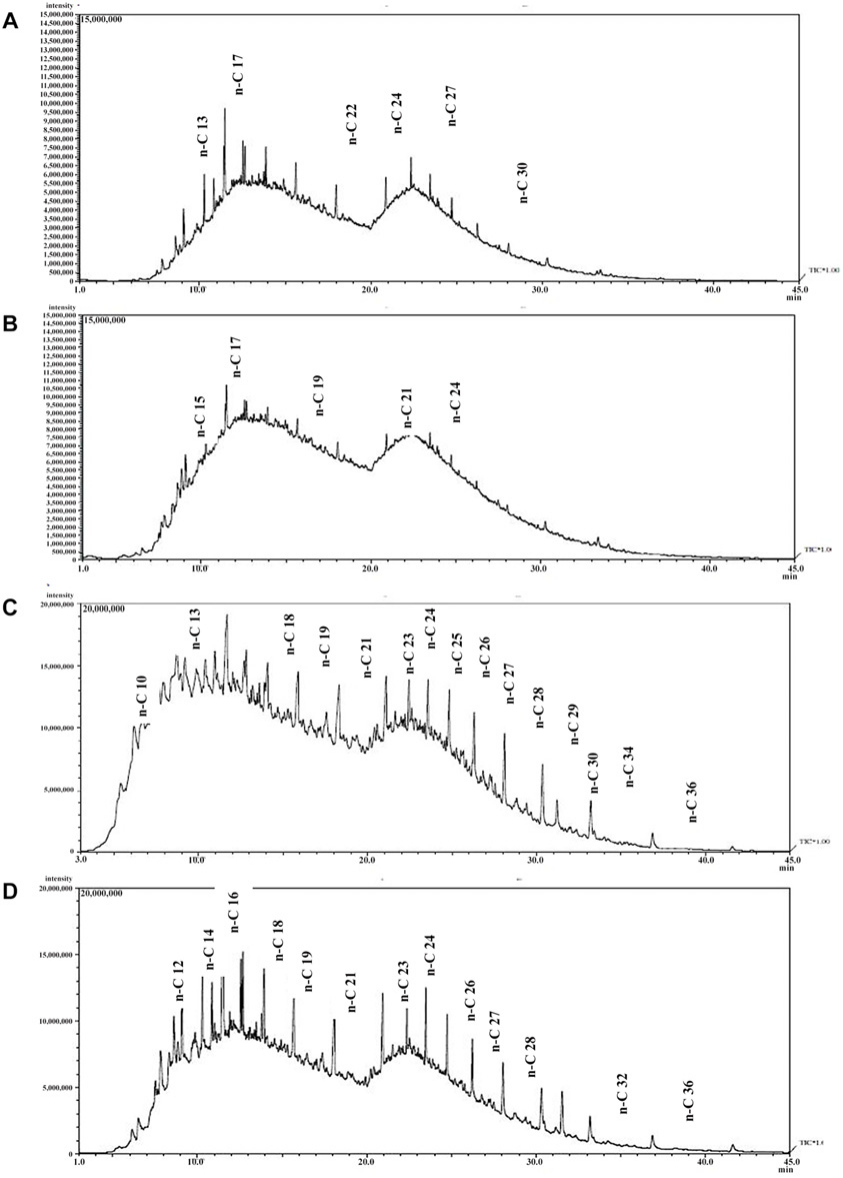
Chromatogram of residual crude oil in polluted water A) with 1% oil, B) With 2% oil, C) control with 1% oil, D) control with 2% oil.

The residual oil was analyzed and identified based on their mass spectra and retention time, as indicated by the chromatogram of the remaining crude oil waste after biodegradation tests (Fig. 7A-D). The results suggest a significant reduction in oil content (C_8_–C_24_) in the bioaugmentation samples compared with the natural attenuation. The effect of strain 139SI on the degradation of the light fraction (C_9_–C_26_) of TPH was higher than the heavy fraction. Low degradation of heavy fractions might be due to the structure of the compounds that make them more complex and strong to break down by the enzyme system of microorganisms [20]. Water polluted with 2% oil there was no complete degradation of fractions and results do not show any significant difference in removing hydrocarbon components in high concentration of crude oil (Fig. 7D). The peaks of long-chain petroleum hydrocarbon were relatively higher than those of short chain hydrocarbons. The reason for the incomplete hydrocarbon fractions biodegradation in high concentration of oil might be attributed to a high poison effect of oil on microorganisms to breakdown the complex structural oil components, which make it significantly difficult for hydrocarbon utilizing bacteria in complete degradation. However, the results are relevant in that the heavy range of hydrocarbon fractions was properly degraded in polluted water with 1% crude oil compared to 2% crude oil and amended with strain 139SI within the period of study. Hydrocarbon components of C_8_–C_14_ fractions are known as the most quickly biodegraded components of oil due to the volatility and their simple structure have been reported. However, they are sensitive to removal by water washing [21].

## Conclusions

Crude oil waste was efficiently degraded by *Bacillus salmalaya* strain 139SI. The bacterial culture was not toxic to strain 139SI during oil degradation. A positive linear correlation occurred between the oil concentration and the rate of biodegradation by *Bacillus salmalaya* strain 139SI. However, the effects of 139SI demonstrated that this bacterium could enhance the water solubility of crude oil waste by approximately 7 times relative to the control.

## Supporting Information

S1 Fig Colony morphology of the novel soil bacteria *Bacillus salmalaya* strain 139SI sheep blood after 24 hours (A) and 48 hours (B) incubation at 37°C. Arrows indicate the areas of complete haemolysis of blood in the medium (β-haemolysis).
(TIF)

S2 Fig Biofilm Inhibitory Concentration test of 139SI against *P. aeruginosa* biofilms coating the glass cover slip of 6-well Microtitter Plat. The lowest concentration of bacterial culture filtrate to inhibit the formation of *P*. *aeruginosa* biofilm on glass cover is highlighted. The X sign indicates that biofilms were not inhibited by 139SI filtrate whereas the highlighted √ sign indictaed a successful biofilm inhibition.
(TIF)

S3 Fig Zone formation in MSM after 20 days of 139SI incubation. (TIF)
