# Supplementary figures and images for: Biotechnological Potential of Bacillus salmalaya 139SI: A Novel Strain for Remediating Water Polluted with Crude Oil Waste
Source: PLoS One. 2015 Apr 13;10(4):e0120931. doi: 10.1371/journal.pone.0120931 (PMC4395392; doi:10.1371/journal.pone.0120931)

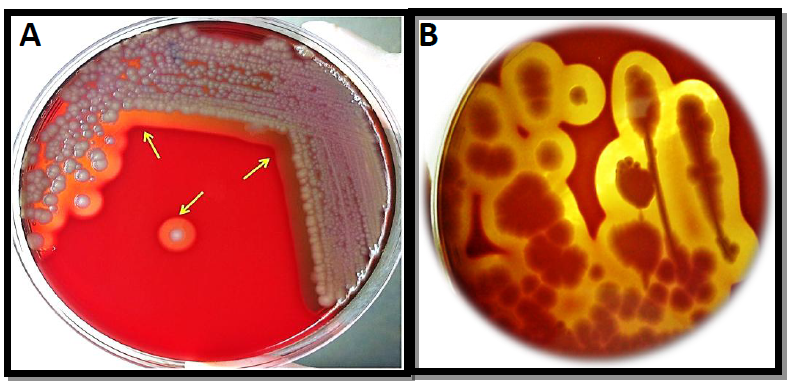

Supplement: S1 Fig — Arrows indicate the areas of complete haemolysis of blood in the medium (β-haemolysis). (TIF) [file pone.0120931.s001.tif]

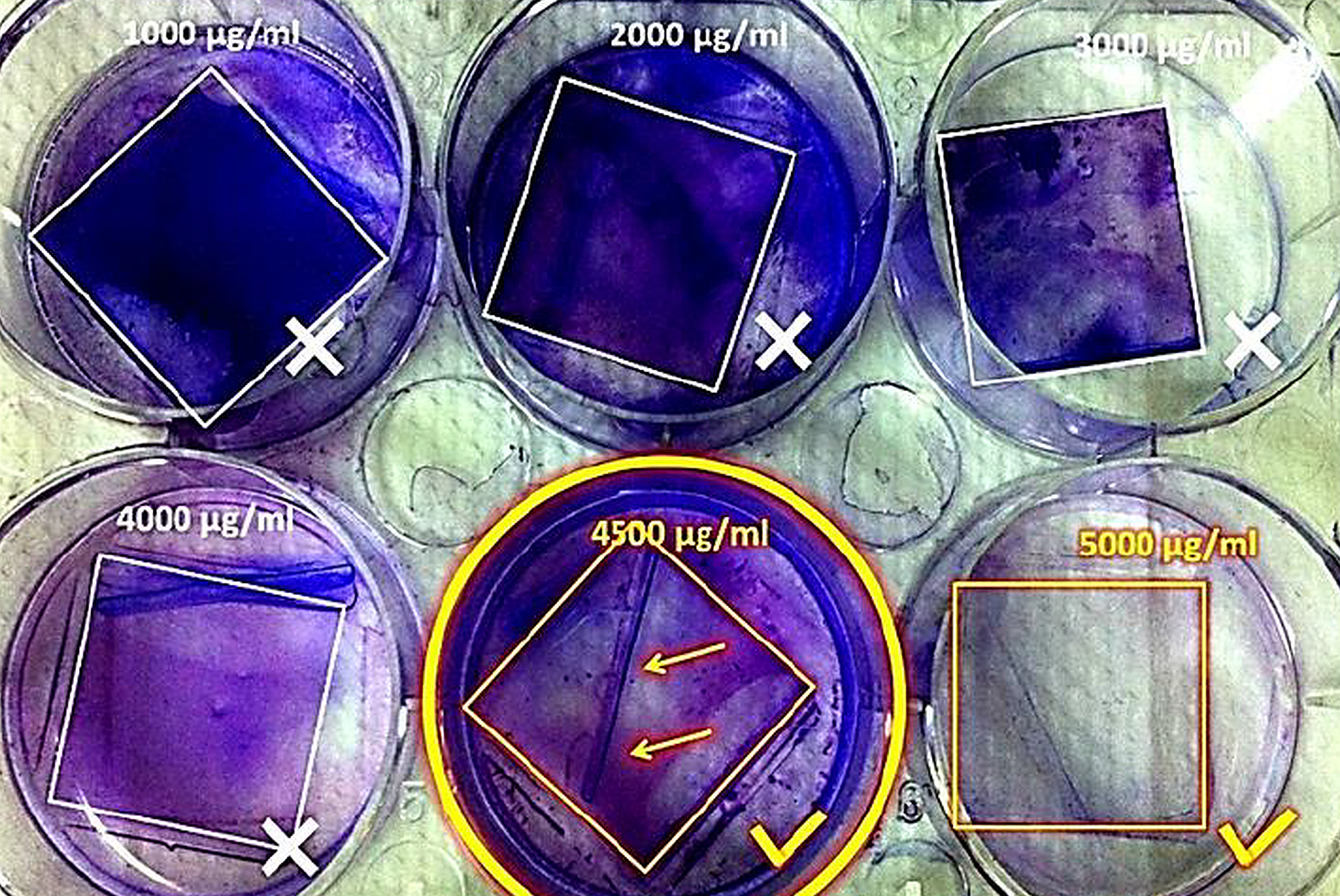

Supplement: S2 Fig — The lowest concentration of bacterial culture filtrate to inhibit the formation of P. aeruginosa biofilm on glass cover is highlighted. The X sign indicates that biofilms were not inhibited by 139SI filtrate whereas the highlighted √ sign indictaed a successful biofilm inhibition. (TIF) [file pone.0120931.s002.tif]

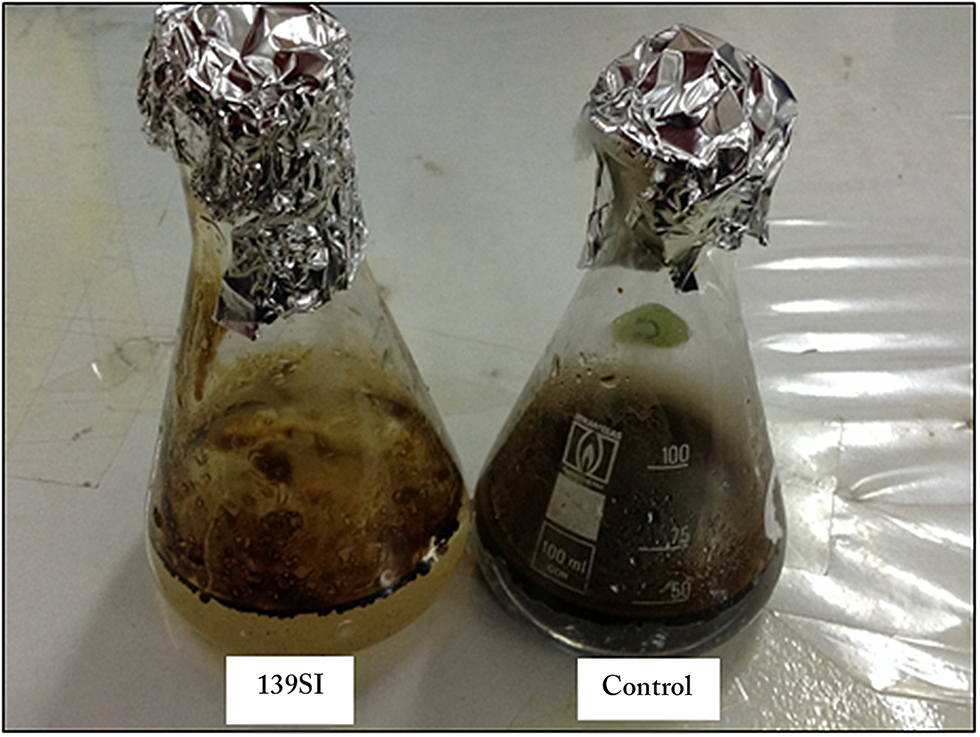

Supplement: S3 Fig — (TIF) [file pone.0120931.s003.tif]
